# Prostate Cancer Units (PCU): A Patients’ Perspective

**DOI:** 10.3332/ecancer.2011.ed10

**Published:** 2011-08-02

**Authors:** Louis Denis

**Affiliations:** Director Oncology Centre Antwerp (OCA), Secretary Europa Uomo, Chair Wij Ook/US TOO Belgium; Lange Gasthuisstraat 35-37, 2000 Antwerp – Belgium

Optimal medical treatment and personalised holistic patient care in an even balance is the ultimate management vision of Europa Uomo, the European prostate coalition, for all patients diagnosed with prostate cancer.

The professional advice and support given on evidence based medicine is the easiest part of the education. We always provide a short but clear overview of the three main prostate diseases: prostatitis, benign prostate hyperplasia (BPH) and prostate cancer (PCa) and their relationship to Prostate Specific Antigen (PSA). The emphasis that we diagnose far more BPH then PCa has an immediate calming effect on the patient concerning ‘abnormal’ PSA results. Extending on the urological impeccable track record on BPH treatment with zero mortality and excellent quality of life outcomes is a good introduction to a prostate cancer diagnosis. The second emphasis on PCa as a complex disease with different biological forms is complimentary information. Concluding with the knowledge that most forms are indolent or low risk as compared to intermediate or high risk disease leads to an optimistic dialogue on prognostic factors and primary treatments of PCa [1].

Some optimism and clarity are needed as our increased knowledge on prognostic factors and the many forms of primary treatment ranging from active surveillance to combination treatments of surgery and radiotherapy make a shared treatment decision a complex issue in the doctor-patient dialogue. There are few facts and many uncertainties which require the expertise of teamwork focused on the individual patient. There is medical and societal consensus that cancer is best treated by a multidisciplinary team confirming the improved outcome results recorded in multidisciplinary clinical trials [2, 3]. The launch and development of Prostate Cancer Units (PCU) as proposed in a discussion paper by the European School of Oncology (ESO) may still improve the outcome results [4].

There are obvious benefits in integrated cancer management for the patients in a one stop shop where a full state-of-the-art program provides not only a consultation with the different experts on your diagnosis and treatment but at the same time access to innovative, tested health technology.

However it is possible that the physician loses some autonomy in turning into a team player with the danger of losing ‘the colloque singulier’, the personal bond of trust between doctor and patient. This loss is already present in our health management as a direct consequence of advanced health technology where the patient has to put blind trust in the hands of an acknowledged expert. It is part of our modern lifestyle when we board airplanes or call for cabs.

Can we save our holistic patient-centered care in the development of PCU? The model should connect intra (hospital) and extra (civil society) mural services. The latter include all professional and lay stakeholders in community health care.

Two factors facilitate the link between medical management and patient care. Health literacy is one of most underrated factors to improve outcomes [5]. It is less easy to adjust the evidence based information to relevance reaching the hidden emotions of patients in different phases of the disease. Next we need a trusted guide in the labyrinth of medical management. The general practitioner takes care of the extramural part while we expect one dedicated professional from the PCU team to be assigned to the patient and the outside guide for relevant, practical and available interactive information.

While we expect our patient responsibilities to facilitate clinical trials, translational research and save scarce health resources we feel that all innovative treatment should report on quality of life and cost-efficiency next to the desired efficacy.

Last but not least we want to emphasize the facilitating role that nursing and patient groups can play between the PCU and community health and social care. The development of PCU´s provides an opportunity for a best practice forum with transparent pathways and outcomes.
